# MRI-Directed Brachytherapy for Cancer of the Uterine Cervix: A Case Report, Review, and Perspective on the Importance of Widespread Use of This Technological Advance in the United States

**DOI:** 10.7759/cureus.15495

**Published:** 2021-06-07

**Authors:** Hiba Z Ahmed, Srinivasan Vijayakumar, William N Duggar, Robert Allbright

**Affiliations:** 1 Radiation Oncology, University of Mississippi Medical Center, Jackson, USA

**Keywords:** locally advanced cervical cancer, definitive treatment, radiation therapy, concurrent chemoradiation, hdr (high dose rate) brachytherapy, 3-d brachytherapy, image-guided brachytherapy, mri-based brachytherapy, local control, pelvic control

## Abstract

Cervical cancer remains a major health challenge in the United States (US), especially among the low socioeconomic and African American populations. The demographics of Mississippi constitute a relatively high percentage of this high-risk population. External beam radiation therapy (EBRT) combined with concurrent chemotherapy and followed by brachytherapy is the gold standard of treatment for stage IB3 through IVA cervical cancer. Arguably, brachytherapy is the most important component of this treatment process. Patterns of Care studies (PCS) and other more recent studies have shown that brachytherapy cannot be omitted or replaced by conventional or image-guided EBRT. The last decade has witnessed the expanding use of image-guided brachytherapy (IGBT). Studies have established the superiority of IGBT over point-based brachytherapy. MRI is associated with superior soft tissue definition compared with CT and is emerging as the new standard of care. The Gynaecological Groupe Européen de Curiethérapie and the European Society for Radiotherapy and Oncology [(GYN) GEC-ESTRO] have recommended that the dose be prescribed to the high-risk clinical target volume (HR-CTV). This volume includes residual tumor present at the time of brachytherapy, the cervix, and any gray areas seen on the scan. The (GYN) GEC-ESTRO has shown that a dose of >8500 cGy delivered in <50 days results in an approximate 10% increase in pelvic control (PC), disease-specific survival, and overall survival (OS) compared to historical controls. The normal tissue toxicity is comparable or better than historical controls as well. This dose, while maintaining normal tissue constraints, may only be achievable with a hybrid intracavitary/interstitial (IC/IS) needle device guided by MRI-based targeting.

The University of Mississippi Medical Center (UMMC) has initiated an MRI-based cervical brachytherapy program and has treated 18 patients to date; our experience confirms the above findings. In this report, we propose that MRI guidance is necessary and a hybrid IC/IS needle device is required to achieve adequate dose coverages.

## Introduction

Cervical cancer is a major medical problem among women, especially women of color and those from lower socioeconomic communities, and the population of the State of Mississippi accounts for a significantly high number of this demographic. The State of Mississippi has the second-highest mortality rate due to cervical cancer in the United States (US), at three per 100,000 women versus two per 100,000 women nationally [1]. Mortality is even higher for African American women in Mississippi, which stands at six per 100,000 women compared to four per 100,000 women for African American women nationally and three per 100,000 women for Caucasian women in Mississippi [1]. This data illustrates the severity of the situation related to cervical cancer among African American women in the State of Mississippi. The outcomes among patients who receive the standard-of-care treatment for this condition are generally good. External beam radiation therapy (EBRT) combined with concurrent chemotherapy and followed by brachytherapy is the standard of care for treating stage IB3 through IVA cervical cancer. The application and technique of brachytherapy is arguably the most important component of this treatment paradigm. Studies have shown that brachytherapy is associated with improved survival outcomes.

Since the 1980s, Patterns of Care studies (PCS) have been published emphasizing that intracavitary (IC) and/or interstitial (IS) radiation therapy is the only treatment-related factor associated with improved pelvic control (PC) and overall survival (OS) in the treatment of cervical cancer [2-7]. The PCS have analyzed and reported on the contemporary practices in the treatment of cervical cancer and identified areas that need improvement. The early PCS established that academic or training facilities, i.e. high-volume centers, were associated with better treatment outcomes [2,3]. Across all stages, the four-year risk of recurrence was 56% when IC brachytherapy was not used compared to 14% with IC. Delving specifically into outcomes for stage IIIB patients, those treated at larger institutions (defined as facilities treating >50 new patients per year) had a four-year PC of 60% versus 28% for patients treated at smaller practices [2]. This discrepancy in PC was attributed to higher point A doses via increased use of IC. Of note, 35% of IIIB patients at small practices did not receive IC versus only 12% of patients treated at large academic institutions. The four-year risk of pelvic failure for stage IIIB in both large and small institutions was 87% when IC was not used. Even when IC was used, only 37% of IIIB patients had poor outcomes at large institutions compared to 63% at small facilities, further emphasizing the importance of physician training, expertise, and patient volume for patient outcomes independent of IC use.

In the early 2000s, there was a trend to replace IC with advanced external beam techniques, such as intensity-modulated radiation therapy (IMRT) and stereotactic body radiation therapy (SBRT), to achieve a similar dose. Han et al. utilized the Surveillance, Epidemiology, and End Results (SEER) database to illustrate that patients with stage IB2-IVA cervical cancer who received brachytherapy after treatment with EBRT had both higher cause-specific survival (CSS) and OS versus those who did not receive brachytherapy [64.3% vs. 52.5% (p<0.001), 58.2% vs. 46.2% (p<0.001), respectively] [8]. Gill et al. obtained similar results using data from the National Cancer Database (NCDB) [9]. Gill et al. were able to demonstrate that the omission of brachytherapy had a greater negative impact on OS than the omission of chemotherapy. Hazard ratios indicated an 86% higher risk of death with the omission of brachytherapy versus a 61% increased risk with the omission of chemotherapy [9].

Over the past three decades, EBRT has undergone a significant transformation, rapidly evolving from two-dimensional (2D) planning in the early phase to three-dimensional (3D) planning and CT-based image-guided radiation therapy (IGRT), and finally to its current modality of MRI-based IGRT. Brachytherapy has witnessed a similar evolution: from 2D point A-based planning to CT point A planning to CT volumetric planning to MRI volumetric planning, pioneered predominately by the Gynaecological Groupe Européen de Curiethérapie and the European Society for Radiotherapy and Oncology [(GYN) GEC-ESTRO]. MRI-based volumetric planning represents a major advance in physicians’ abilities to deliver the high doses needed for superior outcomes while minimizing toxicities, and it is rapidly becoming the gold standard of treatment. This paper aims to highlight the University of Mississippi Medical Center’s (UMMC) efforts to improve outcomes for cervical cancer patients in Mississippi by developing a state-of-the-art MRI-directed brachytherapy program for treating the cancer of the uterine cervix. We describe our one-year experience to emphasize the importance of a careful planning process, multidisciplinary team approach, perseverance in perfecting patient flow processes, and, inevitability, the need for a superb medical physics support.

## Case presentation

We conducted a search of the PubMed database to select articles published between 1990-2020 by using the following keywords: "locally advanced cervical cancer", "definitive treatment", "radiation therapy", "concurrent chemoradiation", "HDR (high dose rate) brachytherapy", "3D brachytherapy", "image-guided brachytherapy", "MRI-based brachytherapy", "local control", "pelvic control", and "survival outcomes".

The results of the implementation of UMMC’s MRI-based brachytherapy program can be exemplified by the case of Ms. CGR. Ms. CGR was a 47-year-old female patient diagnosed with stage IIIB cervical cancer and had been referred to UMMC for the brachytherapy portion of her treatment. She had received the EBRT portion of her concurrent chemoradiation to 45 Gy in 25 fractions and a pelvic sidewall boost of 5.4 Gy in three fractions with weekly cisplatin at an outside facility from October 26, 2020, to December 11, 2020, over the course of 47 days. Her referring physician had first contacted UMMC on December 10, 2020, to discuss Ms. CGR’s case and she was seen for a consult by Radiation Oncology on December 15, 2020, the 51st day since the start of her treatment. She had already received a D90 of 4670 cGy to the high-risk clinical target volume (HR-CTV) from her EBRT. At UMMC, she was treated with four fractions of MRI-based high dose rate (HDR) brachytherapy using a hybrid IC/IS technique, subsequently achieving a final HR-CTV D90 of 8669 in 2-Gy-equivalent fractions (EQD2) cGy.

**Figure 1:**
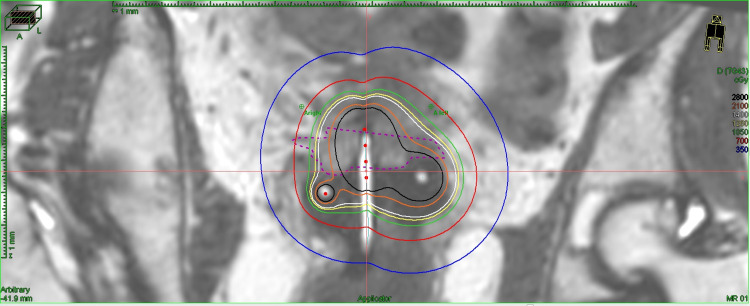
Dose profile for the first fraction on MRI using an IC approach alone MRI: magnetic resonance imaging; IC: intracavitary

At UMMC, the Department of Radiation Oncology in concert with the Departments of Gynecologic Oncology and Radiology has initiated a protocol utilizing MRI planning for each insertion. Images gathered include the T2-weighted non-contrast para-axial, para-sagittal, and para-coronal sequences, a diffusion sequence for lymph node evaluation, and a straight axial 3D space sequence to facilitate contouring by utilizing a Siemens Aera 1.5 Tesla MRI machine (Siemens Medical Solutions Inc. Malvern, PA). At our institution, the device utilized is primarily the Venezia™ (Elekta’s Advanced Gynecological Applicator), a hybrid IC/IS instrument (Elekta Inc., Atlanta, GA). The Venezia™ applicator is utilized without needles for every first fraction, thus serving as a planning template for the use of IS needles during further implants. When IS needles are utilized, up to 10-20% of the total dose is delivered through the needles [10]. The planning system utilized is Oncentra® Brachy (Elekta Inc., Veenendaal, Netherlands). Ultrasound guidance is used at each insertion. MR images are reviewed with a board-certified radiologist for every patient’s, at a minimum, first fraction for tumor/target delineation. The same protocol was used for this patient. Her first insertion used only a tandem with semi-lunar ovoids (T&O) (Figure 1).

This IC-alone approach was only able to deliver 560 cGy to the HR-CTV D90, which equated to 728 cGy EQD2. If an IC approach alone was to be continued, the projected total dose for four fractions of brachytherapy to the HR-CTV D90 from the EBRT and brachytherapy combined would only amount to 7582 cGy (Table 1).

**Table 1:** Projected and actual total doses after the first fraction of brachytherapy* *Outlining the actual total dose to various target volumes and OAR in EQD2 after the first brachytherapy insertion using an IC approach alone and the projected total dose assuming similar doses for the subsequent insertions HR-CTV: high-risk clinical target volume; D2cc: dose to 2 cc; IR-CTV: intermediate-risk clinical target volume; cGy: centigray; OAR: organs at risk; EQD2: equivalent dose in 2-Gy fractions; IC: intracavitary

Planning goals and scorecard				
Metric	Primary	Secondary	Projected total	Actual total
Point A mean >	6500		8649 cGy	5665 cGy
HR-CTV D98 >	7500		6537 cGy	5137 cGy
HR-CTV D90	9000	8500	7582 cGy	5398 cGy
Bladder D2cc	8000	9000	7042 cGy	5158 cGy
Rectum D2cc	6500	7500	7042 cGy	5158 cGy
Sigmoid D2cc	7000	7500	6514 cGy	5026 cGy
IR-CTV D98	6000		6133 cGy	5036 cGy

Even if a D90 of approximately ~700 cGy to the HR-CTV (EQD2 of ~1000 cGy) could be reached with an IC-alone approach to get to a total HR-CTV D90 EQD2 of at least 8500 cGy, the total bladder and rectum EQD2 would have been 8050 and 8160 cGy, respectively, with the rectal dose of 8160 cGy being significantly over the dose tolerance of 7500 cGy (Table 2).

**Table 2:** Projected total doses with IC-alone approach with ~700 cGy per fraction* *Table estimating the total dose to the HR-CTV D90, bladder D2cc, and rectum D2cc in EQD2 assuming each fraction was able to achieve an HR-CTV D90 of ~700 cGy with an IC-alone approach (i.e., assuming ~700 cGy was deliverable to the HR-CTV D90 without the use of needles) HR-CTV: high-risk clinical target volume; D2cc: dose to 2 cc; cGy: centigray; IC: intracavitary; EQD2: equivalent dose in 2-Gy fractions

Metric	Primary	Secondary	Projected total
HR-CTV D90 >	9000	8500	8637 cGy
Bladder D2cc <	8000	9000	8050 cGy
Rectum D2cc <	6500	7500	8160 cGy

**Figure 2:**
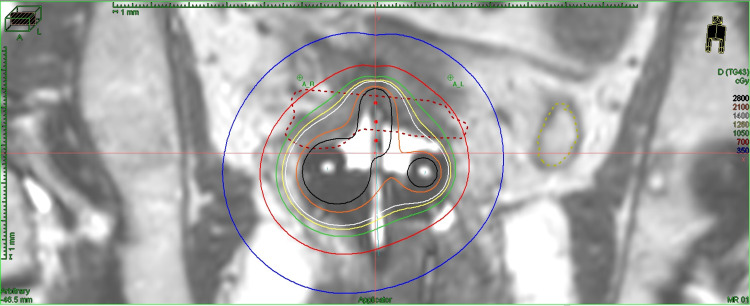
Dose profile of one of the fractions on MRI using an IC/IS approach illustrating the tandem and needles MRI: magnetic resonance imaging; IC/IS: intracavitary/interstitial

**Figure 3:**
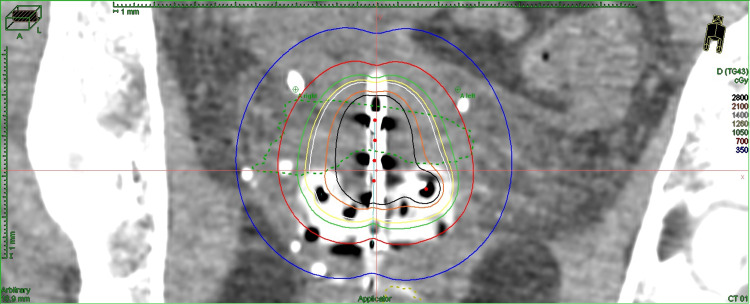
Dose profile of one of the fractions using an IC/IS approach on the planning CT scan illustrating the tandem and needles IC/IS: intracavitary/interstitial; CT: computed tomography

Due to the inability to deliver a sufficient dose while respecting the organs at risk (OAR) through an IC approach alone, the remaining three insertions were done using a hybrid IC/IS instrument (Figures 2, 3).

The subsequent three fractions each delivered a dose over 700 cGy, converted to 1094 cGy, 1083 cGy, and 1094 cGy in EQD2 (Table 3). 

**Table 3:** Doses to target volumes and OAR* *Table listing the actual doses delivered to various target volumes and OAR for each fraction of brachytherapy in EQD2 HR-CTV: high-risk clinical target volume; D2cc: dose to 2 cc; RV: rectovaginal; IR-CTV: intermediate-risk clinical target volume; cGy: centigray; OAR: organs at risk; EQD2: equivalent dose in 2-Gy fractions

Fraction #	One	Two	Three	Four
Fraction date	12/22/20	12/29/20	1/5/20	1/12/21
Point A mean	995 cGy	600 cGy	608 cGy	773 cGy
HR-CTV D98	467 cGy	746 cGy	804 cGy	728 cGy
HR-CTV D90	992 cGy	992 cGy	992 cGy	992 cGy
Bladder D2cc	628 cGy	800 cGy	1050 cGy	963 cGy
Rectum D2cc	628 cGy	749 cGy	724 cGy	651 cGy
Sigmoid D2cc	496 cGy	749 cGy	724 cGy	
RV point dose	470 cGy	360 cGy	430 cGy	420 cGy
IR-CTV D98	366 cGy	352 cGy	338 cGy	437 cGy

Through this method, the patient was able to receive a total dose of 8669 cGy, surpassing the minimum dose recommendation of >8500 cGy while still meeting OAR dose constraints, specifically with a rectal D2cc dose of 7282 cGy, well below the planning goal of <7500 cGy (Table 4). 

**Table 4:** Projected and actual dose after the fourth fraction of brachytherapy* *Table outlining the actual total dose to various target volumes and OAR in EQD2 after the fourth, final brachytherapy insertion (one IC-alone and three IC/IS) HR-CTV: high risk-clinical target volume; D2cc: dose to 2 cc; RV: rectovaginal; IR-CTV: intermediate-risk clinical target volume; cGy: centigray; OAR: organs at risk; EQD2: equivalent dose in 2-Gy fractions; IC/IS: intracavitary/interstitial

Planning goals and scorecard				
Metric	Primary	Secondary	Projected total	Actual total
Point A mean >	6500		7646 cGy	7646 cGy
HR-CTV D98 >	7500		7414 cGy	7414 cGy
HR-CTV D90 >	9000	8500	8637 cGy	8637 cGy
Bladder D2cc <	8000	9000	7972 cGy	7972 cGy
Rectum D2cc <	6500	7500	7282 cGy	7282 cGy
Sigmoid D2cc <	7000	7500	7155 cGy	6499 cGy
RV point dose <	6500	7500	6910 cGy	6910 cGy
IR-CTV D98 >	6000		6163 cGy	6163 cGy

## Discussion

The case detailed above illustrates many salient points regarding the necessity of employing state-of-the-art MRI-directed brachytherapy in the treatment of cancer of the uterine cervix in a rural state, such as Mississippi. At UMMC, the Department of Radiation Oncology in concert with the Departments of Gynecologic Oncology and Radiology has initiated a protocol utilizing MRI planning for each insertion. Thus far, 18 cervical cancer patients have received MRI-based brachytherapy at UMMC. The average HR-CTV volume has been 28.8 cm^3^, and 28% of the cases have required needles (Table 5).

**Table 5:** MRI-based brachytherapy cases at UMMC: tumor and treatment characteristics IC/IS: intracavitary/interstitial; HR-CTV: high-risk clinical target volume; UMMC: University of Mississippi Medical Center; MRI: magnetic resonance imaging

	Number of patients	%	Average HR-CTV (cm^3^)
IC/IS (with needles)	5	28	38.5
IC alone (without needles)	13	72	25.1
Total	18	100	28.8

We have drawn the following conclusions from our experiences:

1. UMMC, the state’s only academic medical center, is the only institution capable of providing MRI-based brachytherapy for this disease currently. Many smaller radiation oncology facilities that are capable of providing EBRT in the state do not have the infrastructure to perform even non-MRI-based brachytherapy. Treating patients who have received their EBRT portion of treatment at an outside facility requires significant medical physics support to obtain Digital Imaging and Communications in Medicine (DICOM)-based data and composite those radiation therapy plans with brachytherapy dose profiles.

2. If we did not have the ability to perform MRI-based brachytherapy, our treatment volumes might have missed areas of viable disease that would not have been apparent on more traditional imaging.

3. If we did not have the hybrid IC/IS brachytherapy applicator, we would have failed to administer sufficient doses to the high-risk volume without exceeding the tolerances of normal tissues.

We will expand further on the above-listed points here.

The evolution of brachytherapy from 2D to 3D-based planning has been illustrated by the Soutien aux Techniques Innovantes et Coûteuses (STIC) Study from France. The STIC Study was a prospective, multi-centric trial designed to compare local/locoregional control outcomes in patients treated with either a 2D or 3D (mostly CT but some MRI) brachytherapy plan [10]. Specifically, patients treated with 3D brachytherapy had better local relapse-free survival (78.5% vs. 73.9%; p=0.003) and locoregional relapse-free survival (69.6% vs. 61.2%; p=0.001) than the 2D cohort. Patients in the 3D cohort developed fewer grade 3-4 toxicities (2.6% vs. 22.7%; p=0.002) [10]. This provides strong evidence that 3D-based dosimetric planning is superior to 2D-based dosimetric planning for brachytherapy.

MRI is associated with superior soft tissue delineation and resolution compared to CT. Moreover, MRI provides a more detailed image of the parametrium and residual disease. Viswanathan et al. prospectively compared dose-volume histograms (DVHs) for the targets and OARs with CT vs. MRI 3D planning [11]. The authors utilized a standardized CT contouring protocol intended to approximate the corresponding (GYN) GEC-ESTRO MRI contouring volumes. The CT volumes consistently overestimated the width of the tumor. MRI-based planning resulted in a greater volume of the HR-CTV treated to the prescription dose or higher. MRI yielded a D100 of 96% versus 86% with CT (p=0.01) [11].

In the early 2000s, (GYN) GEC-ESTRO published recommendations for the acquisition of MRI images to utilize in image-guided brachytherapy (IGBT) [12,13]. The protocol employs T2-weighted non-contrast images of the pelvis. Specific orientations captured include para-axial, para=sagittal, and para-coronal [12]. (GYN) GEC-ESTRO also formulated a series of definitions for the generation of target and OAR DVHs [13]. Gross tumor volume (GTV) has been defined as tumor present at the time of brachytherapy as represented by high signal intensity mass(es) (FSE, T2). The HR-CTV includes the entire cervix, the extra-cervical tumor extension, and any "gray" areas presumed to be related to the disease. The intermediate-risk clinical target volume (IR-CTV) is defined as the HR-CTV plus a 0.5-1.5-cm expansion excluding the bladder and rectum or the original extent of the tumor. Dose recommendations for the D90 HR-CTV, D98 HR-CTV, D98 GTV, D98 IR-CTV, and point A in EQD2 are >85 Gy, >75 Gy, >90 Gy, >60 Gy, and >65 Gy, respectively (Table 6).

**Table 6:** (GYN) GEC-ESTRO planning target goals HR-CTV: high-risk clinical target volume; EQD2: equivalent dose in 2-Gy fractions; GTV: gross tumor volume; (GYN) GEC-ESTRO: Gynaecological Groupe Européen de Curiethérapie and the European Society for Radiotherapy and Oncology

	D90 HR-CTV EQD2	D98 HR-CTV EQD2	D98 GTV EQD2	D98 IR-CTV EQD2	Point A EQD2
Ideal planning goal	>90 Gy, <95 Gy	>75 Gy	>95 Gy	>60 Gy	>65 Gy
Recommended minimum	>85 Gy		>90 Gy		

Dose constraints for the dose to 2 cc (D2cc) of the bladder, rectum, and sigmoid in EQD2 are <90 Gy, <75 Gy, and <75 Gy, respectively (Table 7). A rectovaginal (RV) point was defined as a point 5 mm posterior to the vaginal mucosa at the center of the sources [14]. This serves as a surrogate for estimating late fibrosis of the vagina. EQD2 should ideally be <65 Gy with a secondary acceptable upper limit of <75 Gy as well (Table 7).

**Table 7:** (GYN) GEC-ESTRO OAR planning goals D2cc: dose to 2 cc; EQD2: equivalent dose in 2-Gy fractions; RV: rectovaginal; (GYN) (GYN) GEC-ESTRO: Gynaecological Groupe Européen de Curiethérapie and the European Society for Radiotherapy and Oncology; OAR: organs at risk

	Bladder D2cc EQD2	Rectum D2cc EQD2	Sigmoid D2cc EQD2	RV point dose
Ideal planning goal	<80 Gy	<65 Gy	<70 Gy	<65 Gy
Secondary acceptable maximum	<90 Gy	<75 Gy	<75 Gy	<75 Gy

The (GYN) GEC-ESTRO recommendations listed above were generated from data gathered from a series of sentinel studies as part of a multicenter international study of MRI-based brachytherapy starting in 2008. The first of these studies was image-guided intensity-modulated external beam radiochemotherapy and MRI-guided adaptive brachytherapy in locally advanced cervical cancer (EMBRACE I), which was a prospective single-arm study consisting of patients diagnosed with Fédération Internationale de Gynécologie et d'Obstétrique (FIGO) IB-IVA biopsy-proven squamous cell carcinoma (SCC), adenocarcinoma (AC), or adenosquamous carcinoma (AdSq) of the cervix [15]. Patients were treated with concurrent chemoradiation and MRI-based IGBT [15]. The study aimed to demonstrate superior clinical outcomes of local control (LC), survival, morbidity, and quality of life (QoL) for MRI-based IGBT. This study had accrued 1,416 patients by 2015. Vide infra for results.

Simultaneously, (GYN) GEC-ESTRO initiated a retrospective analysis of patients treated with MRI-based IGBT prior to the initiation of EMBRACE I in 2008; this was the RetroEMBRACE [15]. RetroEMBRACE had three main foci of investigation [16-18]. The first was to illustrate that IGBT combined with concurrent chemoradiation yields improved LC, PC, OS, and CSS compared to 2D brachytherapy historical controls [17]. Secondly, they sought to establish optimal overall treatment time and dose minimums to target volumes [18]. The last goal was to demonstrate that an IC/IS hybrid approach is superior to an IC approach alone for increasing treatment volumes [16]. Vide infra for results.

In 2016, EMBRACE II, another prospective single-arm study, was initiated, which is still undergoing [15]. EMBRACE II represented a further refinement in brachytherapy techniques as advanced by EMBRACE I and RetroEMBRACE. EMBRACE II will attempt to increase QoL by decreasing vaginal fibrosis [19]. One way to achieve this would be to change the weighting of the tandem-to-ring ratio from 50:50 to 66:33. EMBRACE II will also advocate for the increased use of the hybrid IC/IS devices with IS needles, thereby increasing the doses to large tumors and decreasing the ring contribution, which will, in turn, decrease the dose to the RV point. EMBRACE II will also aim to decrease the dose to the vagina via the external beam component by decreasing the overall EBRT volume through "reduced PTV margins, appropriate target selection, systemic contouring, and meticulous treatment planning for IMRT/volume metric modeled arc therapy (VMAT)" [19]. By implementing these changes, it may be theoretically possible to decrease the risk of vaginal stenosis from 21% to 14% [19].

The bulk of the data regarding MRI-based brachytherapy stems from EMBRACE I and RetroEMBRACE. The following data is predominately from RetroEMBRACE. When treated to a dose of at least 85 Gy (D90) delivered in seven weeks, tumors limited in size to 20 cm^3^ had a three-year LC of 94% [18]. Intermediate-sized tumors with volumes between 20-30 cm^3^ had a three-year LC of 93%. Tumors with volumes 31-70 cm^3^ had a three-year LC of 86%. The overall LC was 91%. The mean D90 for the HR-CTV using an IC approach alone was 83 Gy versus 92 Gy for an IC/IS approach (p<0.01). For patients with tumor volumes >30 cm^3^, an IC/IS approach provided a 10% absolute increase in three-year LC [16]. From this data, they were able to extrapolate that for every 10 cm^3^-increase in the volume of tumor size >30 cm^3^, an increase of 5 Gy to the HR-CTV would be required to achieve equivalent levels of LC [18]. Similarly, they were able to extrapolate that for every one-week increase in the overall treatment period beyond seven weeks, again an increase of 5 Gy to the HR-CTV would be required to compensate for that delay in treatment. The three-year PC was 98% for stage IB, 93% for stage IIB, and 79% for stage IIIB [20]. Overall, crude PC was 87% [17]. The overall risk of nodal failure was 11% [21]. Patients who were initially node-positive at diagnosis were more likely to develop nodal failure at 16% versus patients who were initially node-negative at diagnosis, who had a nodal failure of 7%. Nodal failures most commonly occurred at the superior pelvic field border and in the para-aortic region [21]. EMBRACE II will address nodal failure by boosting nodes that are positive at presentation with simultaneous integrated boost (SIB) technique to approximately 60 Gy [19]. EMBRACE II will also divide EBRT patients into small pelvic, large pelvic, and pelvic and para-aortic fields based on varying degrees of nodal positivity at presentation. The actuarial five-year OS for the entire RetroEMBRACE cohort was 65% and five-year CSS was 73% (Table 8) [17].

**Table 8:** Outcomes from RetroEMBRACE and historical controls *Three-year LC. **Five-year DFS. ***Four-year CSS EMBRACE: international study on MRI-guided brachytherapy in locally advanced cervical cancer; NCDB: National Cancer Database; SEER: Surveillance, Epidemiology, and End Results; MRI: magnetic resonance imaging; PC: pelvic control; CSS: cause-specific survival; OS: overall survival; LC: local control; DFS: disease-free survival

	Five-year PC	Five-year CSS	Five-year OS
RetroEMBRACE	84%	73%	65%
EMBRACE I	91%*	Not reported	Not reported
Vale meta-analysis	77%	58%**	66%
UK meta-analysis	78%	59%	55%
NCDB	Not reported	Not reported	54%
SEER	Not reported	64.3%***	54%

Historical controls for patients treated with chemoradiation with 2D brachytherapy include the Vale meta-analysis, the United Kingdom (UK) meta-analysis, SEER, and NCDB data [8,9,22,23]. The Vale meta-analysis analyzed 13 randomized trials worldwide [22]. This data suggested that concurrent chemoradiation improved OS by 6% at five years. The benefit was greater in stage IA and IIA disease than IIIA and IVA disease. PC at five years in RetroEMBRACE was 84% compared to Vale’s 77% (Table 7) [17,23]. The OS for the RetroEMBRACE cohort was 65% at five years [17]. The UK and Vale meta-analyses five-year OS were 55% and 66%, respectively [22,23]. The SEER and NCDB five-year OS were 54 [8,9]. The UK five-year CSS was 59% [23]. The above data indicate a ~10% increase in PC, OS, and CSS for RetroEMBRACE over historical controls.

The American experience with 3D MRI-based brachytherapy is best represented by the University of Pittsburgh study, which accrued patients from 2007 to 2018 [24]. A total of 239 women were studied. The median HR-CTV D90 was 83.7 Gy and the volume was 31 cm^3^. Treatment time averaged 51 days. LC at five years was 90.8% and decreased with AC and HR-CTV >40 cm^3^. Two-year progression-free survival (PFS) was 84.1% for tumors <40 cm^3^ and 66.2% for tumors >40 cm^3^. Two-year OS for tumors <40 cm^3^ was 90.4% while survival for tumors >40 cm^3^ was 65%. Patients with AC, poor response to EBRT, or HR-CTV >40 cm^3^ had inferior LC independent of any increase in doses above 85 Gy. An IC/IS hybrid device was used in 38.9% of the cases [24].

In 2020, The American Society for Radiation Oncology (ASTRO) issued guidelines for the treatment of cervical cancer, including brachytherapy [25]. The recommendations were divided into strong and conditional. ASTRO strongly recommended intra-procedure imaging, e.g., ultrasound, for device placement and MR or CT-based volumetric planning [10,17,25-32]. If this was unavailable, 2D point-based planning was strongly recommended [25,33-37]. ASTRO also strongly recommended total EQD2 of brachytherapy and EBRT to >80 Gy [13,25,30,38]. A D90 ≥8500 cGy was conditionally recommended for tumors that poorly responded to EBRT or tumors >4 cm, with careful consideration to normal tissue constraints [18,39-41]. Also, ASTRO conditionally recommended the utilization of a hybrid IC/IS device to improve dose distribution and to respect OAR constraints. Additionally, ASTRO strongly recommended the use of volumetric planning for OAR, but if it was not available, it strongly recommended 2D point-based constraints [17,25,31,33,34,36,37,40,42-44]. Ideal dose constraints for D2cc of the rectum, bladder, RV point, sigmoid, and bowel in EQD2 were <65 Gy, <80 Gy, <65 Gy (point dose), <70 Gy and <70 Gy, respectively with additional maximum respective dose constraints of: <75, <90, <75 (point dose), <75, and <75 Gy, respectively (Table 9).

**Table 9:** ASTRO OAR dose constraints D2cc: dose to 2 cc; EQD2: equivalent dose in 2-Gy fractions; RV: rectovaginal; ASTRO: American Society for Radiation Oncology; OAR: organs at risk

	Bladder D2cc EQD2	Rectum D2cc EQD2	Sigmoid D2cc EQD2	Bowel D2cc EQD2	RV point dose
Ideal planning goal	<80 Gy	<65 Gy	<70 Gy	<70 Gy	<65 Gy
Secondary acceptable maximum	<90 Gy	<75 Gy	<75 Gy	<75 Gy	<75 Gy

The risk for vaginal stenosis in EMBRACE I was found to be 20% at 65 Gy, 27% at 75 Gy, and 34% at 85 Gy [44]. EMBRACE I also demonstrated a risk of rectovaginal fistula formation at three years of 12.5% with a D2cm^3^ with doses ≥75Gy Gy versus a risk of <2.7% with D2cm^3^ doses <75 Gy [40].

These recommendations are very broad and are intended to be inclusive of practices/institutions with varying degrees of technology. Some practices do not have access to an MRI for treatment planning or possess an IC/IS device. Without IC/IS, it may be very difficult to achieve a dose of 85 Gy with increasing tumor volumes. At EMBRACE institutions that primarily use an IC technique, half of the patients with HR-CTV volumes >30 cm^3^ received a D90 EQD2 dose of <85 Gy [19]. The 80 Gy threshold strongly recommended by ASTRO reflects the results of a trial published by Rao et al., which included 80 patients with stage IIB and IIIB cervical cancer [38]. Patients received 46 Gy EBRT followed by a brachytherapy boost of 600 cGy times four or 800 cGy times three. This resulted in a total EQD2 of 78 or 82 Gy, respectively. They found no significant difference between the two treatment groups. At 30 months, both LC and disease-free survival (DFS) were between 80-90% [38].

We feel that the ASTRO guidelines could have been stronger, which could have helped push policy changes to promote more widespread use of MRI-based brachytherapy for cancer of the cervix. The current bottleneck preventing a more universal use of MRI-based brachytherapy is the lack of investment by even major medical centers. Recommendations made by major cancer policy-making organizations such as the National Cancer Institute (NCI), ASTRO, American Society of Clinical Oncology (ASCO), and American College of Surgeons Commission on Cancer (ACS CoC) can lead to more investment in infrastructure by healthcare facilities and will improve the overall outcomes in cancer of the cervix in the future. Insurance providers also need to consider offering coverage for referral services to MRI-based brachytherapy-capable facilities similar to referrals to proton beam facilities for children with cancer and patients diagnosed with rare tumors such as chordoma of the clivus.

The department has medical physicists who specialize in brachytherapy, and their assistance enables us to perform these complex procedures, as well as the support our institution. The implementation of MRI-based gynecological brachytherapy was planned before its outset and involved multiple disciplines including Gynecologic Oncology, Diagnostic Radiology, Anesthesiology, and Medical Physics. Operational Administration coordinates the patient flow, DICOM image transfers, and the care, safety, and convenience of patients and their families.

In a very recent publication, the risk for vaginal stenosis from EMBRACE I was reported to be 20% at 65 Gy, 27% at 75 Gy, and 34% at 85 Gy [44]. EMBRACE I also demonstrated a risk of RV fistula formation at three years of 12.5% with a D2cm^3^ with doses ≥75 Gy versus a risk of <2.7% with D2cm^3^ doses <75 Gy [40]. Most recently, EMBRACE I published its results on risk factors and dose effects for bladder fistula and cystitis. They found that the crude incidence rate for bladder fistula greater than grade 1 was 0.7% and that an increase from 75 Gy to 80 Gy in bladder D2cm^3^ resulted in an increase from 8% to 13% of cystitis greater than grade 1 [45].

## Conclusions

In this report, we highlighted the importance of using MRI-based brachytherapy in the treatment of cancer of the uterine cervix to improve outcomes and the necessity of adopting a team approach in implementing and operationalizing such a program. The benefits of such a program can enable the delivery of adequate doses to the tumor volumes without exceeding the dose tolerances of the normal tissues. Finally, we cannot overemphasize the need for a careful multidisciplinary approach to carry out this complex procedure safely for the benefit of our patients.
